# Tolerability of breast ductal lavage in women from families at high genetic risk of breast cancer

**DOI:** 10.1186/1472-6874-9-20

**Published:** 2009-07-14

**Authors:** Jennifer T Loud, Ellen Burke Beckjord, Kathryn Nichols, June Peters, Ruthann Giusti, Mark H Greene

**Affiliations:** 1 Clinical Genetics Branch, National Cancer Institute, Rockville, MD, USA; 2 Associate Behavioral and Social Sciences Researcher, RAND Corporation, Pittsuburgh, PA, USA; 3 Westat, Inc, Rockville, MD, USA; 4 Center for Biologics Evaluation and Research, Food and Drug Administration Rockville, MD, USA

## Abstract

**Background:**

Ductal lavage (DL) has been proposed as a minimally-invasive, well-tolerated tool for obtaining breast epithelial cells for cytological evaluation of breast cancer risk. We report DL tolerability in *BRCA1/2 *mutation-positive and -negative women from an IRB-approved research study.

**Methods:**

165 *BRCA1/2 *mutation-positive, 26 mutation-negative and 3 mutation unknown women underwent mammography, breast MRI and DL. Psychological well-being and perceptions of pain were obtained before and after DL, and compared with pain experienced during other screening procedures.

**Results:**

The average ***anticipated ***and ***experienced ***discomfort rating for DL, 47 and 48 (0–100), were significantly higher (*p *< 0.01) than the ***anticipated ***and ***experienced ***discomfort of mammogram (38 and 34), MRI (36 and 25) or nipple aspiration (42 and 27). Women with greater pre-existing emotional distress experienced more DL-related discomfort than they anticipated. Women reporting DL-related pain as worse than expected were nearly three times more likely to refuse subsequent DL than those reporting it as the same or better than expected. Twenty-five percent of participants refused repeat DL at first annual follow-up.

**Conclusion:**

DL was anticipated to be and experienced as **more **uncomfortable than other procedures used in breast cancer screening. Higher underlying psychological distress was associated with decreased DL tolerability.

## Background

Ductal lavage (DL) is a method of collecting breast epithelial cells from the lining of the breast duct by means of a small-gauge catheter inserted into a ductal orifice on the nipple to permit direct access to exfoliated breast duct epithelial cells. Information gained from DL might improve early breast cancer detection, facilitate breast cancer risk assessment, and yield novel reagents for developing biomarkers and intermediate end-points in chemoprevention trials [1]. Epithelial atypia in cells collected from nipple aspirate fluid (NAF) and by random peri-aereolar fine needle aspiration (RPFNA) has been associated prospectively with an increased risk of non-invasive and invasive breast cancer [2-5]. It was hypothesized that women with cellular atypia in DL samples would also be at increased breast cancer risk. However, obtaining adequate numbers of cells from DL samples for both cytological review and biomarker development has been a challenge [6]. Reliably obtaining NAF from all or most women studied, and acquiring samples with cell counts adequate for cytologic evaluation (> 10 evaluable cells) from DL specimens has been problematic [1,7-19]. Neither NAF production nor 5-year Gail risk > 1.7% [6,20,21] predicted atypia in DL specimens from high-risk women [9,21]. It is possible to detect atypia in both NAF-yielding and non-NAF-yielding ducts from women at high-risk of breast cancer [6,7,9,12]; however, it is not known whether the atypia detected by DL will demonstrate an increased prospective risk of breast cancer in women at high genetic risk of breast cancer, and there is increasing evidence that reproducibility of cytologic diagnoses in benign duct epithelial specimens and in specimens with atypia found on DL is only fair-to-poor [6,11,13,17]. Furthermore, if DL is to be clinically useful, it is essential that the procedure be well-tolerated, or healthy women will not comply with its use.

There are several reports of DL-related pain in women at high risk of breast cancer, but few women who were known to be *BRCA1/2 *mutation carriers, and even fewer who were unaffected *BRCA1/2 *mutation carriers, were included in these reports [1,13,18,19]. Visual analogue scales [1,13,18,19] or Likert-type scales [19] were used to assess DL-related pain and were administered immediately after the procedure. The variability in the statistical methods used for the analyses makes it difficult to compare the findings across the groups. Two groups reported that DL was well-tolerated [1,18], one group reported that DL was not well-tolerated [13] and a fourth group reported that there was more maximal discomfort reported with DL than with mammogram or breast MRI [19].

Emotional distress might influence DL tolerability, since previous general population studies have suggested that women reporting higher levels of pre-procedure distress experience greater mammogram-related discomfort [22-24]. Importantly, unpleasant mammogram-related experiences have been associated with decreased likelihood of returning for annual breast cancer screening [24,25]. However, previous studies of DL tolerability [1,13,18,19] have neither assessed emotional distress nor analyzed its influence on DL tolerability.

The National Cancer Institute Clinical Genetics Branch Breast Imaging Study (BIS; NCI Protocol 01-C-0009) is a four-year, prospective cohort study of 200 women from families with known *BRCA1 *or *BRCA2 *mutations. *BRCA1/2 *mutation carriers have an estimated lifetime risk of breast cancer between 45%–82% [26-29]. Chemoprevention is often considered for breast cancer-risk reduction [30-32] and annual mammography and breast MRI are employed in the early detection of breast cancer among high-risk women not choosing risk-reducing mastectomy [33-38]. The overall research goal of the BIS was to improve early detection of precursor and malignant breast lesions by evaluating several breast screening procedures: annual DL, mammography, breast MRI, NAF and clinical breast exam. As the study progressed, we recognized that DL seemed more painful than previously described. We report these results, compare pain from DL with other breast screening procedures, and describe associations between DL pain, participant characteristics, and acceptance of annual DL.

## Methods

### Study Population

Eligible women were between ages 25–56, **and **had a known deleterious *BRCA1/2 *mutation, **or **were first- or second-degree relatives of *BRCA1/2 *mutation carriers, **or **were first- or second-degree relatives of individuals with *BRCA-*associated cancers in *BRCA1/2 *mutation-positive families. Exclusion criteria included: pregnancy or lactation within 6 months of enrollment, abnormal CA-125, bilateral breast cancer, ovarian cancer or breast cancer (Stage IIB or greater), unless relapse-free for 5 years prior to enrollment. Participants with a personal history of DCIS, or Stages I/II breast cancer were eligible, provided that ≥ 6 months had elapsed since completing primary therapy. Other exclusion criteria included: a personal history of other invasive cancer (except for non-melanoma skin cancer), unless relapse-free for 5 years prior to enrollment; prior bilateral mastectomy; bilateral breast irradiation; weight > 136 kilograms; and gadolinium allergy. DL was not performed on participants with allergy to lidocaine or bupivacaine; peri-areolar or other breast surgery which might disrupt the ductal systems of the breast; a breast implant or prior silicone injections in the breast; and active infection or inflammation in the breast to be studied.

### Participants

This analysis includes data from 194 women who were enrolled during the period June 2002 through May 2006. Reasons for not performing DL included physician cancellation (n = 13), being ineligible (n = 4), and refusing (n = 5). Participants who did not attend both pre- and post-DL clinic visits were excluded from the analysis (n = 38). Excluded women were similar to those analyzed relative to procedure tolerability ratings, sociodemographic variables, and other study outcomes, except that excluded women were more likely to have a history of breast cancer and to have had NAF obtained during DL (*p *< 0.05). The final study sample included 156 (194 – 38) participants.

Mean participant age was 39.4 (*SD *= 8.6); 90% had attained > high school education. Eighty-five percent were *BRCA1/2 *mutation-positive, and 10% reported prior breast cancer. Ninety-seven percent of subjects were white, a proportion reflecting the Clinical Genetics Branch referral pattern.

### Procedures

Participants were ascertained from: the NCI-DCEG Familial Cancer Registry (41/156; 26%) and various healthcare providers (99/156; 64%), primarily in response to mailed recruitment letters, or were self-referred from our BIS website 16/156 (10%) [39]. The protocol was reviewed and approved by the NCI Clinical Center IRB; written informed consent was obtained from all participants.

### Ductal Lavage Protocol

**Figure 1:**
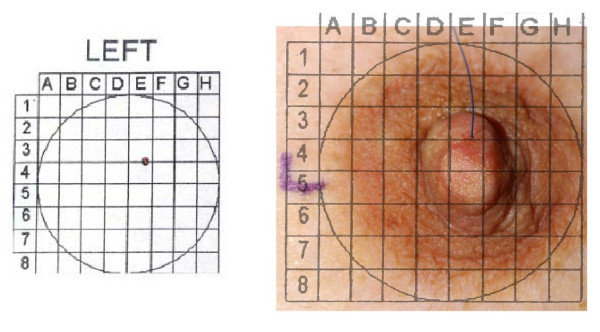
**Documentation of DL Procedure**.

Standard preparation of the breast, including topical anesthetic (4% lidocaine cream) applied to the nipple/areola 60 minutes prior to the procedure, was provided to all DL participants. The nipple/areolar complex surface was gently probed with a micro dilator tip prior to DL, to confirm adequate anesthesia. In women who reported probing-related pain, additional topical anesthesia and/or subcutaneous injections of lidocaine around the base of the nipple were administered. Nipple aspiration was performed to identify all fluid-yielding ducts. We attempted to identify and cannulate all visible ducts, regardless of NAF status (Cytyc Health Corporation, Boxborough MA; Acueity, Palo Alto, CA). After successful catheter insertion, 3–5 mL of 1% lidocaine was infused, followed by 20 mL of sterile normal saline, in 5 mL aliquots. After each aliquot was infused, the breast was massaged and fluid collected *via *the lavage catheter. The location of each lavaged duct was recorded by threading a blue suture into the duct orifice, photographing the breast (Figure 1) and storing the photo in the participant's permanent medical record.

Three clinicians performed the BIS DL procedures: JTL, adult nurse practitioner, RAG, medical oncologist and LR, surgical oncologist. The clinicians were trained to perform ductal lavage using the technique described by Dooley et al [1]. Most frequently, DL was performed by two clinicians working together, alternating between right and left breast. There was no indication that tolerability changed during the 4 years required to accrue patients, as the clinicians acquired more experience performing ductal lavage. The preparation of the breast through the completion of the ductal lavage required approximately one hour for each breast studied.

### Measures

#### Procedure tolerability

Four indices of breast screening procedure tolerability were used.

First, women rated each procedure's (mammogram, MRI, NAF, and DL) discomfort on a 0 to 100 scale, with 100 indicating the worst discomfort imaginable. On Day 1, participants rated *anticipated *discomfort *before *undergoing any of the screening procedures, and on Day 2 participants rated *experienced *discomfort *after *completing all screening procedures.

In addition, participants were asked on Day 2 whether discomfort associated with each screening procedure was worse, the same as, or better than expected.

Women were queried whether the DL-associated discomfort was worse, the same, or better than 7 common medical procedures (flu shot, blood draw, pelvic examination, mammogram, chest x-ray, Novocaine shot, rectal exam), and

Participants characterized pain quality by selecting between the descriptors burning, aching, radiating, sharp, dull, fullness, or tingling.

#### Emotional distress

The Brief Symptom Inventory 18 (BSI 18) Global Severity Index (GSI) was used to quantify overall psychological distress levels. The BSI 18 has been widely used to assess distress in medical oncology settings, with good reliability and validity [40]. It was administered in clinic before participants underwent screening procedures and assessed their levels of distress *on that day*.

#### Follow-up DL Screening

Women were asked to return for follow-up DL screening one year after initial screening. Participants either completed follow-up DL, cancelled their appointment, refused follow-up DL, or had not received follow-up DL at the time of the current analysis for another reason (had yet to be scheduled, missed follow-up appointment, left the study, or became ineligible for follow-up DL due to partial mastectomy, diagnosis of breast cancer, or pregnancy).

#### Sociodemographic and medical variables

Prior reports have examined age, presence of NAF, history of breast cancer and *BRCA1/BRCA2 *mutation status in relation to DL tolerability; therefore, these variables were included in the present study [1,13,18,19].

### Data analyses

Data analyses were conducted using SPSS Version 15.0 (2006). Bivariate analyses were used to 1) compare continuous ratings of both anticipated and experienced procedure discomfort (0–100) between DL and other breast screening procedures, using paired *t*-tests, and 2) to examine continuous ratings of experienced DL discomfort in relation to study variables (age, history of breast cancer, *BRCA1/BRCA2 *mutation status, whether NAF was obtained during DL, GSI, and whether participants returned for follow-up DL) using bivariate correlation (*r*), independent samples *t*-tests, or ANOVA. Multivariate analyses used linear and logistic regression to model continuous ratings of anticipated and experienced DL discomfort, and to estimate the odds of refusing follow-up DL. Some participants declined one or more of the screening procedures; therefore, sample size is reported for each analysis presented in the Tables.

## Results

**Figure 2:**
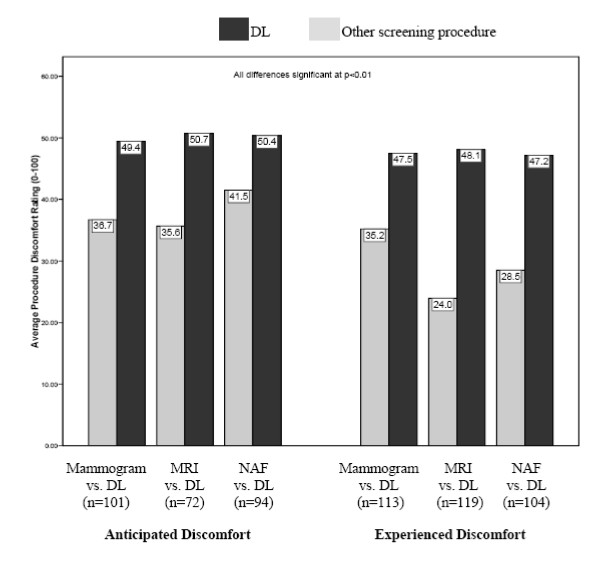
**Paired *t*-tests comparing screening procedure discomfort ratings (0–100) (higher scores indicate greater discomfort)**.

MRI and NAF were significantly less painful than anticipated (*p *< 0.05); mammogram and DL ratings of experienced discomfort did not differ significantly from participants' anticipation (Table 1). Figure 2 shows anticipated and experienced discomfort of DL compared with other study screening procedures for women who completed both. In all cases, DL discomfort was anticipated to be, and experienced as, significantly worse than mammogram-, MRI-, and NAF-related discomfort (all *p *< 0.01), suggesting that in this cohort, DL was tolerated significantly less well than other breast screening procedures. The majority of women described the discomfort associated with DL as "sharp" (78%) or "burning" (52%). The majority of women also reported that DL discomfort was the same as/worse than discomfort associated with a Novocaine injection (65%), a blood draw (61%), a pelvic (59%) or rectal examination (64%).

**Table 1:** Paired *t-tests *for anticipated and experienced discomfort with breast cancer screening procedures (n = 156)

**Study Variable**	**Type of Data**	**Sample percent or Mean (*SD***)	**P-value**
Anticipated mammogram discomfort (n = 103)	Continuous†	36.8 (*18.4*) (median = 30)	*p *= .388
Experienced mammogram discomfort (n = 146)	Continuous†	34.4 (*23.3*) (median = 30	
Anticipated MRI discomfort (n = 74)	Continuous†	36.3 (*17.6*) (median = 30)	*p *= **0.023**
Experienced MRI discomfort (n = 153)	Continuous†	24.9 (*23.7*) (median = 20)	
Anticipated NAF discomfort (n = 94)	Continuous†	41.5 (*20.1*) (median = 40)	*p *= **0.00**
Experienced NAF discomfort (n = 126)	Continuous†	27.1 (*24.0*) (median = 20)	
Anticipated DL discomfort (n = 145)	Continuous†	47.2 (*20.4*) (median = 50)	*p *= 0.392
Experienced DL discomfort (n = 120)	Continuous†	47.7 (*23.9*) (median = 50)	

†Range for continuous measure of discomfort for all breast screening procedures = (0–100); higher scores indicate greater discomfort.

Bivariate analyses (Table 2) indicated that neither age, *BRCA1/BRCA2 *mutation status, nor whether NAF was obtained during DL were associated with experienced DL discomfort. Women with a prior breast cancer rated DL experiences as marginally *less *painful than women without such a history (*p *= 0.05). Higher levels of distress were associated with significantly higher ratings of experienced DL discomfort (*p *< 0.05), with women who refused follow-up DL reporting the highest experienced DL discomfort (*p *< 0.01).

**Table 2:** Bivariate associations between DL tolerability and study variables†

		**Mean rating of experienced DL discomfort (*SD*) or bivariate correlation (*r*)**	***p***
Age	Continuous	-0.12	0.18
Personal history of breast cancer	Yes	35.0 (*18.3*)	**0.05**
	No	49.1 (*24.1*)	
*BRCA1/2 *mutation status	Positive	47.8 (*23.6*)	0.90
	Negative	47.0 (*26.7*)	
NAF obtained during procedure	Yes	47.7 (*25.6*)	0.99
	No	47.7 (*23.4*)	
BSI GSI	Continuous	0.20	** < 0.05**
Returned for follow-up DL	Yes	40.5 (*19.5*)	** < 0.01**
	Refused	61.7 (*25.7*)	
	Cancelled	45.9 (*25.4*)	
	Other	45.8 (*22.4*)	

†Range for continuous measure of discomfort for all breast screening procedures = (0–100); higher scores indicate greater discomfort.

Multivariate methods were used to examine anticipated and experienced DL discomfort ratings (Table 3), and odds of returning for follow-up DL (Table 4). In these analyses, age, breast cancer history, whether NAF was obtained during DL, and GSI scores were entered into the regression equation in one step. For the linear model of experienced DL discomfort, anticipated discomfort was entered as a second step (data for the final model are shown). The categorical indicator of experienced DL discomfort was included in the logistic model of odds of refusing follow-up DL. Because of concerns related to sample size, and the lack of a significant bivariate association, *BRCA1/2 *mutation status was not included in the multivariate models.

**Table 3:** Multivariate linear regression models for discomfort associated with DL

**Multivariate linear** **regression**	**Type of Data**	**Anticipated DL discomfort** **(n = 122) Model R^2 ^= 0.00**		**Experienced DL discomfort** **(n = 95) Model R^2 ^= 0.08**	
**Step 1**		β(SEβ)	*p*	β(SEβ)	*P*
Age	Continuous	0.14 (0.22)	0.53	-0.33(0.30)	0.27
History of breast cancer	Yes No	-0.45 (6.15) *Ref*	0.94	-10.79 (8.01) *Ref*	0.18
NAF obtained during procedure	Yes No	2.79 (4.55) *Ref*	0.54	2.19 (5.79) *Ref*	0.71
BSI GSI	Continuous	0.11 (0.20)	0.59	0.57 (0.27)	** < 0.05**
**Step 2**					
Anticipated DL discomfort	Continuous	*n/a*	*n/a*	0.09 (0.12)	0.48
BSI GSI	Continuous	*n/a*	*n/a*	0.50 (0.28)	0.08

None of the study variables were associated with ratings of anticipated DL discomfort (Table 3). For experienced discomfort, higher levels of distress (measured by the GSI) were associated with ratings of greater experienced DL discomfort (β = 0.57; *p *< 0.05) in Step 1. Adding anticipated discomfort to the model in Step 2 did not change the results of Step 1; anticipated DL discomfort was not associated with experienced DL discomfort (*p *= 0.48), although the effect for GSI became marginally significant (*p *= 0.08) when anticipated discomfort was added to the model.

We used multivariate logistic regression to model the odds of refusing follow-up DL (24% of participating women refused) (Table 4). Women with NAF during DL had marginally lower odds of refusing follow-up DL (OR = 0.3; *p *= 0.06). Higher scores on the GSI (indicating greater emotional distress) were associated with higher odds of refusing follow-up DL (OR = 1.1; *p *< 0.01). Women who reported that DL discomfort was worse than they expected it to be (34% of the study sample) were more than three times more likely to refuse follow-up DL (OR = 3.2; *p *= 0.03) than those reporting DL discomfort as the same, or better than expected (66% of participants). Results were consistent in a second regression model in which we substituted the continuous experienced DL discomfort measure (0–100) for the categorical discomfort classifier. In the continuous model, the odds of refusing follow-up DL increased by 5% with every 1-point increase in experienced DL discomfort ratings (*p *< 0.01; data not shown).

**Table 4:** Multivariate logistic regression model of returning for DL

**Multivariate logistic regression**	**Type of Data**	**Odds of refusing DL** **(vs. Returned/** **Cancelled/Other)(n = 98)**	
		**OR (95%CI)**	***p***
Age	Continuous	1.00 (0.94,107)	0.93
History of breast cancer	Yes No	0.26 (0.03, 2.63) *Ref*	0.26
NAF obtained during procedure	Yes No	0.26 (0.06, 1.08) *Ref*	0.06
BSI GSI	Continuous	1.08 (1.02, 1.15	**0.01**
Experienced DL discomfort	Worse Same/better	3.19 (1.11, 9.12) *Ref*	**0.03**

## Discussion

In this group of women from families at high genetic risk of breast cancer, experienced DL-related discomfort was significantly worse than has been reported previously. The anticipated and experienced discomforts of DL were significantly greater than the anticipated and experienced discomforts related to other common medical procedures and breast cancer screening methods. The majority of women described their DL-associated pain as "sharp" or "burning." Women reporting DL-related pain as worse than expected were nearly three times more likely to refuse subsequent DL than those reported it as the same or better than expected. Women who reported high ratings of pre-study distress (as measured by the BSI-18) were more likely to experience greater DL-related discomfort than women with lower distress ratings.

Several findings have direct impact on the management of women undergoing DL. First, it seems apparent that for most women undergoing DL, the current anesthesia protocol is inadequate. We were able to achieve acceptable anesthesia at the nipple surface, but passing the catheter through the constricted ductal sphincter was an important source of procedure-related pain. Local anesthesia alone did not relax this sphincter, and nor did using topical nitro-paste (for smooth muscle relaxation) make a significant difference.

It is somewhat surprising that younger women and women without a prior history of breast cancer had similar reports of discomfort with DL when compared with older women and women with a prior history of breast cancer. Compared with other groups who have reported on DL tolerability [1,13,18,19], our cohort contained greater numbers of mutation carriers, more young women, and fewer women with a prior history of breast cancer. Although we did observe marginally better reported DL tolerability among women with a history of breast cancer in bivariate analyses, the small number of women with a prior history of breast cancer in this cohort may have limited our ability to demonstrate a statistically significant difference in DL tolerability between affected and unaffected participants in regression analyses.

Pain associated with DL has been evaluated by several groups [1,13,18,19]. Dooley et al., reported on 507 women who had DL performed on at least one breast; 291 had a prior history of breast cancer, 10 had a history of lobular carcinoma *in situ*, 199 were high risk due to a Gail Model risk of ≥ 1.7, 4 were not at high risk of breast cancer, and only 3 were *BRCA1/2 *mutation carriers. A median pain score of 28 mm on a 0–100 mm visual analogue scale which was administered immediately after DL was reported (1). However, 28% of subjects underwent the procedure in the operating room under general anesthesia and less than one percent of the subjects were cancer-unaffected, known *BRCA1/2 *mutation carriers. No comparison of pain scores between subjects who received general anesthesia *versus *those who did not was reported.

Mitchell et al. (20) employed a visual analogue scale (range 0–10) to record measurements of pain in 52 women with *BRCA1/2 *mutations. A similar rating of 2.8/10 was reported immediately after DL, and DL pain was described as similar to the pain experienced with mammography. As with the previous study (1), more than 50% of participants were breast cancer survivors. Neither group reported whether differences existed in measures of pain between women with a prior history of breast cancer and unaffected women. It is possible that women who are breast cancer survivors experience pain differently from women without a prior history of breast cancer. Neither group (1,18) reported the acceptance rate of future DL in their study populations.

DL tolerability was reported in a retrospective study in women at high risk of breast cancer who had been evaluated as part of a breast cancer screening study [19]. Twenty-two *BRCA1/2 *mutation carriers rated DL-related pain on a scale of 1 to 3 (1 = minimal discomfort, 2 = moderate discomfort, 3 = maximal discomfort), and compared their experience with DL to breast MRI on a scale of 1 to 5 (1 = much better, 2 = somewhat better, 3 = same, 4 = somewhat worse, 5 = much worse). *BRCA1/2 *mutation carrier participants more often rated DL as maximally uncomfortable versus MRI or mammogram, and the maximal discomfort ratings for DL vs. mammogram and MRI combined reached statistical significance (P = 0.04). There was no difference in reports of pain between breast cancer survivors and unaffected women; however, the sample size was small (breast cancer survivors, n = 13; unaffected, n = 23), and mutation status was not reported. Future acceptance of DL was not reported in this study population.

The reliability and acceptability of DL in 69 women at high risk of breast cancer due to Gail Model score ≥ l.66 (n = 38), a family history of breast cancer (n = 53), the presence of a *BRCA1/2 *(n = 2), a personal history of abnormal breast biopsies (atypical hyperplasia, non-invasive or invasive breast cancer, n = 20) or a prior history of breast cancer (DCIS/invasive breast cancer, n = 11) was found to be less than ideal [13]. A visual analogue scale from 0–10 was employed to measure DL pain at visit one and six months later at the second visit. The mean pain score at visit one was 4 (range, 0–8) and the mean pain score at visit 2 was 3 (range, 0–9). After visit one, 70% of the women who underwent DL reported that they would have DL again, and if recommended, would undergo the procedure as part of routine early breast cancer detection. However, only 52% of these women returned for a second visit. There were insufficient numbers of known *BRCA1/2 *mutation carriers within this group of women to determine whether mutation carriers differ in measures of pain from other women at high risk of breast cancer.

Emotional distress might influence measures of DL pain and acceptance, since previous general population studies have suggested that women describing higher levels of emotional distress report greater mammogram-related discomfort [22-24]. Furthermore, unpleasant mammogram-related experiences have been associated with decreased likelihood of returning for annual breast cancer screening [24,25]. However, previous studies of DL tolerability [1,13,18,19] have neither assessed emotional distress nor analyzed its influence on DL pain and acceptance.

### Study Strengths

To date, this is the largest group of women with known *BRCA1/2 *carrier mutation status to undergo annual DL. It is also the largest group of *unaffected *mutation carriers which has undergone DL as part of an annual breast cancer screening program. All procedures were performed by the same 3 experienced clinicians. Furthermore, this represents the first formal assessment of emotional distress as a modifier of DL tolerability, and these data were collected using a widely-applied, clinically-validated psychometric tool, the BSI-18. All indices of tolerability were defined prior to enrollment of the first subject, and the analytic plan was stipulated in advance. Therefore, the study provides robust, high-quality information regarding the feasibility of adding DL to current breast cancer screening strategies for women at high genetic risk of breast cancer. Our data indicate that poor DL tolerability poses a significant barrier to its more widespread clinical application.

## Limitations

The Breast Imaging Study is a single-institution intervention study of highly-selected women. Women who participated in this study are both highly-educated and highly-motivated to participate in clinical research. It is unlikely that this group of women is representative of all women from *BRCA1/2 *mutation-positive families or other healthy women in the general population who are at high risk of breast cancer. Therefore, our findings may not apply to a more general population of *BRCA1/2 *mutation carriers nor other women at high risk of breast cancer. However, we doubt that the presence of these highly-selected women in a study of DL tolerability specifically designed for woman from *BRCA1/2 *mutation-positive families biased the findings. Although a small proportion (15%) of participants had taken part in multiple prior NCI HBOC-related clinical research projects [41], the vast majority were first-time enrollees in a CGB study.

## Summary

Our experience suggests that DL is not likely to play a central role in breast cancer screening among high-risk women, because women find it painful, are reluctant to undergo multiple DL examinations over time, and because the procedure has also failed to yield large enough numbers of exfoliated epithelial cells from high-risk women to permit reliable cytologic diagnosis or to support translational research activities [7,13]. In addition, DL is time and personnel intensive; the average time required to perform bilateral DL was 2 hours and required two clinicians (MD or NP, plus an assistant). Finally, the biological plausibility of attempting early diagnosis/risk stratification on the basis of sampling 1 or 2 ducts from a 20 duct system has not been persuasive. We have discontinued this procedure as a routine component of our ongoing BIS research protocol.

## Conclusion

In conclusion, DL was introduced into clinical practice only nine years ago; consequently, systematic prospective studies of its tolerability are limited. Regardless of its ultimate utility as a diagnostic, risk assessment or research tool, for its use to be practical, it is essential that DL be well-tolerated. Despite vigorous attempts to obtain satisfactory local anesthesia (short of doing the procedure in the operating room), nearly 25% of our participants refused to repeat the DL one year later.

The current study highlights the important relationships between measures of emotional distress, experience of DL discomfort and refusal of subsequent DL. Women who report high levels of emotional distress just prior to DL are three times more likely to refuse DL in the future. Additionally, women who were more distressed were more likely to report poor DL tolerability, and poor tolerability was associated with less acceptance of future DL. The identification of women who are emotionally distressed near the time of DL provides an opportunity to address the distress directly, either by using anxiolytic medications, rescheduling the appointment to a less stressful time, or referral for emotional counseling prior to DL.

The identification of the quality of pain, e.g., sharp, burning, allows for refinement in the anesthesia of the breast nipple. Conscious sedation may be required for optimal pain relief in women who report emotional distress prior to DL or in women who experience significant levels of pain during DL. This, of course, would substantially increase both the cost and logistical difficulty of applying this tool in the clinic.

Our experience [17] and others [7] suggests that alternative strategies aimed at breast cancer early detection, risk stratification and acquisition of tissue for translational research in high-risk women are required. Multiple core biopsies of the breast is an option, but one with which there is neither widespread experience nor ready acceptance by either institutional review boards or patients, particularly if multiple samples over time are required. However, this technique has been used successfully as a one-time-only specimen collection strategy in a breast cancer chemoprevention trial [42]. At present, the leading candidate for such an alternative is RPFNA of the breast, as pioneered by Fabian and colleagues [3].

## Competing interests

The authors declare that they have no competing interests.

## Authors' contributions

JTL drafted the manuscript, conceived of the study and participated in the study design, procedure performance and data collection. EBB performed the data analyses, drafted the results section of the manuscript and contributed to the development of the manuscript. KN coordinated all data and specimen collection. EBB's work on this manuscript was completed while in the Cancer Prevention Fellowship in the Divisions of Cancer Prevention and Cancer Control and Population Sciences at the National Cancer Institute. JP participated in the study design and in the manuscript preparation. RG participated in the specimen collection, study design and manuscript preparation. MHG provided senior leadership for the study, funding and participated in the manuscript development. All authors read and approved the final manuscript.

## Pre-publication history

The pre-publication history for this paper can be accessed here:


